# Exploring Therapeutic Potentials and Pathways of Psychedelics in Alzheimer’s Disease‐Related Dementia

**DOI:** 10.1002/alz.084413

**Published:** 2025-01-09

**Authors:** Jitendra Kumar Sinha, Shampa Ghosh, Krishna Kumar Singh, M P Singh, Manchala Raghunath

**Affiliations:** ^1^ GloNeuro Academy, Noida, Uttar Pradesh India; ^2^ ICMR ‐ National Institute of Nutrition, Hyderabad, Telangana India; ^3^ Symbiosis Centre for Information Technology, Pune, Maharastra India; ^4^ Graphic Era (Deemed to be University), Dehradun, Uttarakhand India

## Abstract

**Background:**

Traditionally associated with recreational and spiritual uses, psychedelics have gained attention in psychotherapy for their therapeutic potential. Functioning as potent 5‐hydroxytryptamine (5HT) agonists, these compounds have demonstrated the ability to enhance neural plasticity by activating serotoninergic and glutamatergic systems. Despite these recognized effects, their role in treating neurodegenerative disorders, particularly dementia, remains relatively unexplored.

**Method:**

Recent studies have unveiled modulatory and beneficial impacts of psychedelics, including N, N‐dimethyltryptamine (DMT), Lysergic acid diethylamide (LSD), and Psilocybin, in Alzheimer’s disease (AD)‐related dementia. These compounds target neurotransmitter imbalances and molecularly modulate AD‐related signaling pathways, such as the Brain‐derived neurotrophic factor (BDNF) pathway, mTOR activation, and other autophagy regulators.

**Result:**

The controlled administration of psychedelics, contingent on dosage, presents a novel therapeutic intervention for AD‐related dementia, warranting exploration for drug development. This review critically examines existing literature on the therapeutic potential and pathways of psychedelics for AD‐related dementia.

**Conclusion:**

While promising, further studies are imperative to establish long‐term safety, efficacy, and optimal treatment protocols. Integrating psychedelics into the treatment paradigm may offer a transformative approach to address the unmet needs of individuals with AD‐related dementia and their caregivers.

